# Host Response to *Staphylococcus epidermidis* Colonization and Infections

**DOI:** 10.3389/fcimb.2017.00090

**Published:** 2017-03-21

**Authors:** Thuan H. Nguyen, Matthew D. Park, Michael Otto

**Affiliations:** Pathogen Molecular Genetics Section, Laboratory of Bacteriology, National Institute of Allergy and Infectious Diseases, National Institutes of HealthBethesda, MD, USA

**Keywords:** coagulase-negative staphylococci, *Staphylococcus epidermidis*, innate immunity, host defense, sepsis, biofilms, biofilm-associated infection

## Abstract

The majority of research in the *Staphylococcus* field has been dedicated to the understanding of *Staphylococcus aureus* infections. In contrast, there is limited information on infections by coagulase-negative Staphylococci (CoNS) and how the host responds to them. *S. epidermidis*, a member of the coagulase-negative Staphylococci, is an important commensal organism of the human skin and mucous membranes; and there is emerging evidence of its benefit for human health in fighting off harmful microorganisms. However, *S. epidermidis* can cause opportunistic infections, which include particularly biofilm-associated infections on indwelling medical devices. These often can disseminate into the bloodstream; and in fact, *S. epidermidis* is the most frequent cause of nosocomial sepsis. The increasing use of medical implants and the dramatic shift in the patient demographic population in recent years have contributed significantly to the rise of *S. epidermidis* infections. Furthermore, treatment has been complicated by the emergence of antibiotic-resistant strains. Today, *S. epidermidis* is a major nosocomial pathogen posing significant medical and economic burdens. In this review, we present the current understanding of mechanisms of host defense against the prototypical CoNS species *S. epidermidis* as a commensal of the skin and mucous membranes, and during biofilm-associated infection and sepsis.

## Introduction

Coagulase-negative staphylococci (CoNS) are a heterogeneous group of staphylococcal species classified clinically by the absence of the blood-clotting enzyme coagulase. This distinguishes them from *Staphylococcus aureus* and a few clinically less important coagulase-positive species. Today, CoNS are the most commonly isolated bacteria in clinical cultures and have emerged as major nosocomial pathogens. Risk factors for CoNS infection include the presence of indwelling medical implants, such as intravascular catheters, or immunosuppression due to cancer treatment or HIV/AIDS. Treatment of CoNS infections is complicated by the emergence of antibiotic-resistant strains (such as particularly MRSE, methicillin-resistant *S. epidermidis*) (Rogers et al., 2009).

CoNS are an integral part of the normal flora on the human skin and mucous membranes, and preferentially colonize moist areas (Grice et al., 2009). *S. epidermidis*, the most common CoNS species recovered from clinical cultures, colonizes the armpit, groin, anterior nares, conjunctiva, toe webs, and perineal area (Kloos and Musselwhite, 1975). While usually innocuous or even beneficial colonizers, once the host epithelial barrier is compromised, CoNS such as *S. epidermidis* can cause serious infections. In fact, CoNS infections account for the majority of bacterial sepsis and foreign body-related infections, with *S. epidermidis* being the most significant species in that regard (Rogers et al., 2009).

The host immune response to *S. epidermidis*, the mechanism of immune tolerance, and the immune benefits that *S. epidermidis* commensals can provide, are just beginning to be unraveled. This review will provide the latest research on the host response to *S. epidermidis* as commensals, and as opportunistic bacteria in the context of biofilm and septic infections.

## The host immune response to *S. epidermidis* as a commensal

There is increasing evidence that the skin microbiota in general have an important impact on the immune system (Belkaid and Tamoutounour, 2016). Despite CoNS being among the most important skin colonizers, specific studies on the host immune response to CoNS colonization and establishment have been limited. Those that are available have focused on the immune response to skin colonization by the prototypical CoNS species *S. epidermidis* and the benefits such colonization provides to the host.

**Figure 1:**
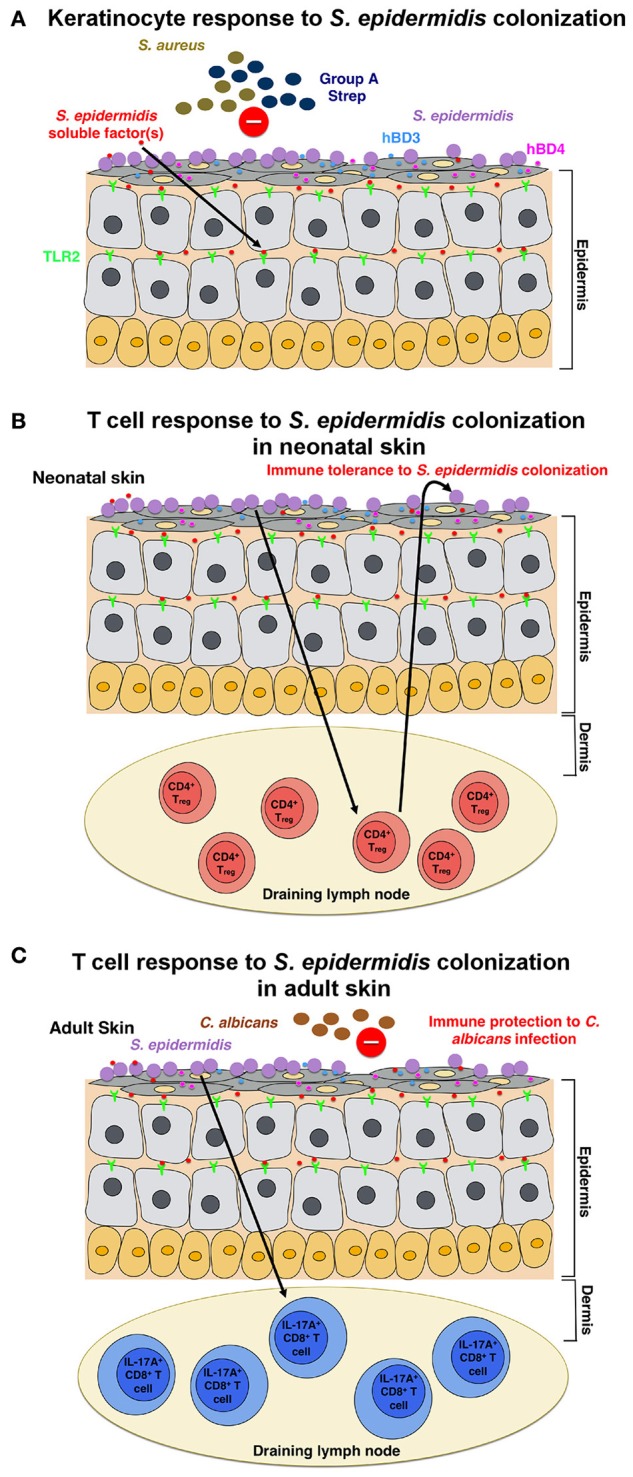
**Model of the host response to *S. epidermidis* colonization (A)** *S. epidermidis* colonizes the skin epidermis, a highly organized structure composed mainly of keratinocytes. In response to *S. epidermidis* colonization, pattern recognition receptors (PRRs) such as TLR2 on keratinocytes bind to poorly characterized factor(s) secreted by *S. epidermidis* to stimulate a signaling cascade that results, for example, in the production of the antimicrobial peptides β-defensin 2 (hBD2) and hBD3. These antimicrobial peptides provide protection from cutaneous *S. aureus* and Group A *Streptococcus* infections. **(B)** *S. epidermidis* colonization induces a specific CD4^+^ FOXP3^+^ T_reg_ response, which is essential for immune tolerance toward *S. epidermidis* as a commensal. Immune tolerance is believed to be established only during the neonatal period, as colonization in adult mice failed to establish tolerance. **(C)** In a different model, based on results obtained in adult mice, skin colonization by *S. epidermidis* triggers a specific IL-17A^+^ CD8^+^ T-cell response. Primed in the skin draining lymph node by CD103^+^ dendritic cells, these T cells enhance the innate antimicrobial defense and prevent invasion by the fungus, *C. albicans*.

For example, the Gallo group has described potentially beneficial functions of *S. epidermidis* as a skin commensal. Namely, Lai et al. reported that *S. epidermidis* lipoteichoic acid (LTA) through a mechanism involving TLR (toll-like receptor) 2 reduces skin inflammation (Lai et al., 2009). The same authors also later reported that *S. epidermidis* (but not other bacteria) produces a not further characterized substance of less than 10 kD that activates TLR2, and thereby induces antimicrobial peptide production, which increased the capacity of cell lysates to inhibit growth of group A *Streptococcus* and *S. aureus* (Lai et al., 2010) (Figure 1A). While these results underline a beneficial function of *S. epidermidis* on the skin, regarding the stimulating factor, they have to be seen in light of the fact that the frequently reported activation of TLR2 by LTA has been challenged: LTA purification is extremely difficult and often contains TLR2-stimulating lipopeptide contaminants, including in commercial preparations (Hashimoto et al., 2006). These findings thus certainly require further assessment using isogenic mutants of *S. epidermidis* to verify the nature of the stimulating factor.

Scharschmidt et al. showed that colonization with *S. epidermidis* triggered a local, as well as systemic, specific CD4^+^ T cell response as demonstrated by the enrichment of specific CD4^+^ T cells in both the skin-draining lymph nodes and the spleen (Scharschmidt et al., 2015). This group engineered the *S. epidermidis* skin isolate, strain Tü3298, to express the peptide antigen 2W (Epi-2W) linked to a fluorescent protein. To achieve colonization, they applied 10^8^–10^9^ CFUs of the engineered Epi-2W strain to the dorsal skin of C57BL/6 mice every 3 days for a total of three applications. Using this model, the authors illustrated that expansion of specifically CD4^+^ regulatory T (T_reg_) cells plays a critical role in the immune tolerance to *S. epidermidis*. Such tolerance, however, required early bacterial colonization during the neonatal stage, as colonization in adult mice did not establish tolerance (Figure 1B). The work by Scharschmidt et al. is especially intriguing as it provides the scientific rationale for the modulation of the skin microbiota in the neonatal period as a therapeutic option to treat inflammatory skin diseases, and in particular, for atopic dermatitis (AD).

The skin of AD patients is often colonized by *S. aureus* (Higaki et al., 1999). Several studies show that *S. epidermidis* is the second most common microbe isolated from AD-affected skin lesions (Hon et al., 2005, 2012, 2016). While this does not directly implicate *S. epidermidis* in the pathogenesis of AD, due to its normal and frequent abundance on the skin, in the most recent of those studies, Hon *et al*. examined bacterial isolates from 100 AD patients and found that *S. epidermidis* is present in the most severely AD-affected skin lesions (Hon et al., 2016). Thus, the previously suggested antagonistic relationship between *S. epidermidis* and *S. aureus* (Cogen et al., 2010; Iwase et al., 2010), did not translate to less disease in their study. Rather, the results suggested that *S. epidermidis* colonization is associated with more severe AD disease. Certainly, further studies are required to evaluate the role of *S. epidermidis* in AD pathogenesis and the nature of the relationship between *S. aureus* and *S. epidermidis* in AD lesions.

Naik et al. demonstrated that *S. epidermidis* colonization in adult mice induces a skin-specific T cell response (Naik et al., 2015). In this study, the group applied ~5 ml of 10^7^–10^9^ CFU per ml of *S. epidermidis* across the entire mouse skin surface every other day for a total of four applications, a procedure by which they reported to obtain stable colonization. The T cells induced in this study, however, were IL-17A^+^ CD8^+^ T cells rather than CD4^+^ T cells, as in the Scharschmidt et al. study (Scharschmidt et al., 2015). The authors showed that these IL-17A^+^ CD8^+^ T cells provided immunity to cutaneous *C. albicans* infection (Figure 1C); however, they did not examine whether this also provides immunity to other pathogens, notably *S. aureus* (Naik et al., 2015).

Both the Scharschmidt et al. and Naik et al. studies examined the host immune response to *S. epidermidis* colonization, yet their findings are quite different. These differences can be attributed to the nuances in the setup of the mouse models or possibly also the specific *S. epidermidis* strains used. Despite the differences in the results, collectively, the two studies showed that colonization with *S. epidermidis* induces an adaptive T cell response in mice. It is unclear, however, if such immune signature to *S. epidermidis* colonization is also observed in humans.

## Immunity against *S. epidermidis* in biofilm-associated infections

Very few studies have been performed to assess the immune response to CoNS biofilm-associated infections, especially as compared to *S. aureus*. Most of those studies have focused on *S. epidermidis*. CoNS biofilm-associated infections often occur in patients with medical implants, with *S. epidermidis* being the most likely species to be recovered (Rogers et al., 2009). Biofilms are complex, spatially diverse agglomerations of bacterial cells enclosed within an amorphous, self-produced extracellular matrix composed of extracellular DNA, proteins, and polysaccharides (Otto, 2008). Biofilm-associated infections are extremely resistant to antibiotic treatment for several reasons, including reduced metabolism and hampered penetration through the extracellular matrix (Mah and O'Toole, 2001). Consequently, treatment often requires surgical removal of the infected device or tissue. In the US alone, approximately $ 2 billion is spent annually for the treatment and management of orthopedic implant-related infections (Darouiche, 2004; Parvizi et al., 2010).

**Figure 2:**
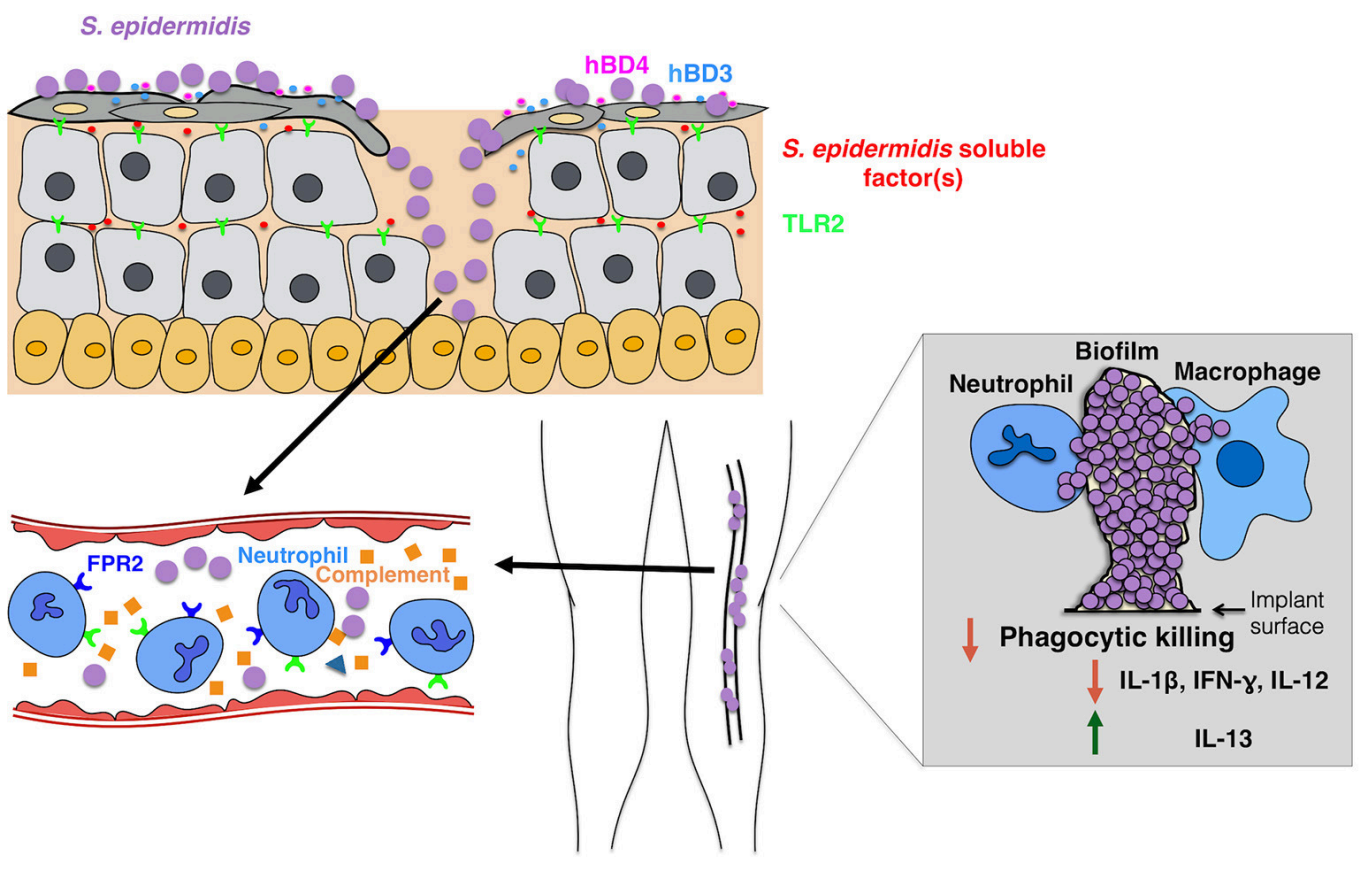
**Model of the host response to *S. epidermidis* infections**. Breaching through the skin can result in *S. epidermidis* dissemination into the bloodstream to cause bacteremia and acute cases of sepsis. Host immunity against septic infections heavily depends on neutrophils, complement, as well as activation of PRRs including G-protein coupled receptors (GPCRs) such as TLR2 (green) and FPR2 (blue). Biofilm-associated infections on medical implants originate from contaminations during device insertion or, in rare cases, from the bloodstream. Patients with medical implants are susceptible to *S. epidermidis* sepsis, as biofilm bacteria can often disseminate into the bloodstream. The immune response to biofilm-associated infections is generally thought to be not effective, as biofilms inhibit phagocytic killing by PMNs and macrophages. In addition, they skew the immune system toward enhanced production of anti-inflammatory cytokines such as IL-13 while limiting the secretion of pro-inflammatory cytokines, including IL1-beta, IL-12, and IFN-gamma.

In general, the host immune response to an *S. epidermidis* biofilm-associated infection is not protective or sufficient to clear the infection. Hence, most *S. epidermidis* biofilm-associated infections are chronic. Most studies conducted on the subject thus far are *in-vitro* studies that have compared biofilm-grown versus planktonic bacteria. Results from several studies suggest that *S. epidermidis* biofilms induce attenuation in phagocytic function and production of anti-inflammatory cytokines when compared to their planktonic counterparts (Figure 2). Conflicting findings have been reported on the effects of *S. epidermidis* biofilms on phagocytic activity. Two studies reported that adherence and phagocytosis by human neutrophils and primary human monocyte-derived macrophages were significantly enhanced when stimulated with biofilm-grown bacteria (Heinzelmann et al., 1997; Spiliopoulou et al., 2012), while others reported that the biofilm exopolysaccharide, polysaccharide intercellular adhesin (PIA) (Mack et al., 1996) plays an essential role in the attenuation of phagocytic capacity of murine peritoneal macrophages (Shiau and Wu, 1998), J774A.1 murine macrophages (Schommer et al., 2011), and human PMNs. There is consensus in the literature, however, with regard to the effects of *S. epidermidis* biofilm on phagocytic killing. Killing by human macrophages (Spiliopoulou et al., 2012) and PMNs (Vuong et al., 2004; Kristian et al., 2008) as well as antibody-mediated killing by leukocytes (Cerca et al., 2006) are strongly attenuated in the presence of biofilm-grown bacteria.

The role of complement in CoNS biofilm-associated infections is quite unclear. While *S. epidermidis* biofilm-producing strains elicit a stronger response in the activation and release of complement components than their isogenic PIA-negative and thus biofilm-negative counterparts (Kristian et al., 2008; Fredheim et al., 2011), such complement release did not translate to enhanced phagocytic killing. *S. epidermidis* PIA-positive biofilms triggered C3a release, but protected *S. epidermidis* from C3b and IgG opsonization and PMN-mediated killing (Kristian et al., 2008). Notably—while some authors tried to attribute specific effects to PIA, rather than biofilm formation, by mechanically destroying aggregates (Vuong et al., 2004), in none of these studies a clear distinction between the effects of biofilm agglomerations and a direct effect of the exopolysaccharide PIA can be made. As for the claimed pro-inflammatory properties of PIA (Kristian et al., 2008; Fredheim et al., 2011; Ferreirinha et al., 2016), the facts that PIA is a difficult-to-purify substance and isogenic PIA-negative mutants have distinctly different cell surface properties, makes it difficult to attribute observed effects directly to the PIA molecule.

It has been reported that *S. epidermidis* biofilm-grown strains elicit production of anti-inflammatory rather than pro-inflammatory cytokines (Spiliopoulou et al., 2012). In the respective study, primary human monocyte-derived macrophages stimulated with live *S. epidermidis* from 24-h biofilms produced lower levels of pro-inflammatory cytokines (IL-1beta, IFN-gamma, IL-12) and elevated levels of the anti-inflammatory cytokine IL-13 than planktonic cells grown for 2 h. Obviously, in that comparison other factors, such as most notably quorum-sensing-regulated pro-inflammatory factors such as the phenol-soluble modulins (PSMs) (Cheung et al., 2014) discussed below, may be made responsible for the observed differences rather than biofilm formation itself. However, comparing biofilm-positive with isogenic biofilm-negative (PIA-negative, Embp, or Aap-negative) strains, Schommer et al. also observed a reduced inflammatory response in 774A.1 macrophages with reduced NF-kappaB activation and reduced IL-1beta production (Schommer et al., 2011).

Little is known about how the adaptive immune system responds to biofilm-associated infections, in part, because it is difficult to establish long-term *S. epidermidis* biofilm infection models. Vuong et al. have developed a catheter-related murine infection model with a *S. epidermidis* bioluminescent strain called SE Xen43, with which they were able to monitor in real-time the progression of *S. epidermidis* biofilm-associated infection (Vuong et al., 2008). Comparing the susceptibility of Nu/Nu (T cell-deficient) and CBSCBG-MM (T/B cell-deficient) to *S. epidermidis* biofilm-associated infection with immuno-competent wild-type Balb/C mice, the authors found that in particular the Nu/Nu mice were more susceptible to infection, indicating an important role of T cell-mediated immunity against *S. epidermidis* biofilm-associated infection.

## Immunity against *S. epidermidis* during sepsis

The presence of CoNS in the blood (bacteremia), often originating from the dispersal of bacteria from biofilms on indwelling medical devices, can cause acute sepsis (Figure 2). CoNS bacteremia is associated with significant healthcare costs, morbidity, and mortality (Bearman and Wenzel, 2005). Immune-compromised and premature neonates are the most vulnerable to CoNS sepsis with *S. epidermidis* being the most prevalent CoNS species involved (Cheung and Otto, 2010).

In contrast to *S. epidermidis* biofilm-associated infections, which are chronic, *S. epidermidis* sepsis is acute by nature. Therefore, the host immune response to *S. epidermidis* sepsis, which takes place largely in the bloodstream, is quite different from that against tissue-residing *S. epidermidis* biofilm associated-infections. Since neonates have increased susceptibility to *S. epidermidis* sepsis, most of the studies on the matter dealt with *S. epidermidis* neonatal sepsis. Therefore, the following discussion will be focused on neonatal immunity in response to S. *epidermidis* sepsis.

An important element in the innate immune response are innate immune cells, among which neutrophils dominate in number. Neutrophils recognize invading microbes via a repertoire of host receptors (see below), ingest them, and eliminate them within the phagosome by reactive oxygen species and antimicrobial proteins released during a process called degranulation (Malech et al., 2014). In addition, lysed neutrophils can form neutrophil extracellular traps (NETs) to bind and kill invading microbes (Brinkmann et al., 2004).

Several pattern recognition receptors (PRRs), which recognize and bind to conserved microbial products (PAMPs, pathogen-associated molecular patterns) and play an essential role in the activation of the innate immune response, have been shown to be critical in host immunity against *S. epidermidis* sepsis. TLR2 was shown to be critical for clearance of *S. epidermidis* in a mouse sepsis model (Strunk et al., 2010). In human neonates, there is an increase in TLR2 expression over the course of *S. epidermidis* sepsis (Viemann et al., 2005), however, a TLR-stimulated immune system was reported to be less proficient in eliciting multiple cytokine responses in neonates compared to adults (Kollmann et al., 2009). *S. epidermidis* PIA (Stevens et al., 2009), PSMs (Hajjar et al., 2001), and lipoteichoic acid (LTA) (Xia et al., 2016) have been claimed to be effectors of TLR2. However, studies with PIA and PSMs were not verified with isogenic mutants; and as for LTA, there has been recent evidence indicating that staphylococcal lipopeptides rather than LTA are the real immune-stimulatory agents (Hashimoto et al., 2006). Furthermore, in *S. aureus* it was shown that PSMs are not direct agonists of TLR2, but lead to the release of lipopeptides from the cell surface and thus have a secondary, TLR2-stimulatory effect (Hanzelmann et al., 2016), an effect likely also present in *S. epidermidis*.

Based on the fact that the formyl peptide receptor 2 (FPR2) recognizes PSMs (Kretschmer et al., 2010), this G protein-coupled chemoattractant receptor is another potentially important host receptor in the response against *S. epidermidis*. As shown in *S. aureus*, PSM-FPR2 activation induces chemotaxis, granule exocytosis, and interleukin-8 (IL-8) release from PMNs (Wang et al., 2007; Kretschmer et al., 2010). Studies on the effects of PSMs of CoNS on the immune response have only been performed with pure PSMs of *S. epidermidis* (Cheung et al., 2010), and so far have been hampered by the multitude of genetic *psm* loci in *S. epidermidis* and the general difficulty to produce isogenic deletion mutants in CoNS. However, a recent study attributed a crucial role to the mobile genetic-element-encoded, highly produced PSM-mec of *S. epidermidis* in inflammation and immune evasion, using isogenic *psm-mec* mutants (Qin et al., 2017).

Complement also plays an important role in the immunity against *S. epidermidis* sepsis. Deficiencies in complement factor C3 and IgG are associated with a higher risk of neonatal CoNS-associated sepsis (Lassiter et al., 1991). Furthermore, in a study using an *ex-vivo* whole-blood sepsis model, *S. epidermidis* induced significantly lower complement activation in neonatal compared to adult blood (Granslo et al., 2013). This finding suggests that there is a maturational deficiency in the neonatal complement system, which, in part, may explain why neonates are more susceptible to *S. epidermidis* septic infections than adults. Moreover, this study highlights the importance of the complement system in the host defense against *S. epidermidis* sepsis.

Like complement, neutrophils of preterm neonates display maturational deficiency. They show an impaired oxidative burst compared to those isolated from term newborns when stimulated with *S. epidermidis* (Björkqvist et al., 2004). This, in part, may also explain the increased susceptibility in preterm neonates to *S. epidermidis* sepsis. Interestingly enough, monocytes, another cellular component in the innate immune response, are not essential to the host defense against neonatal *S. epidermidis* sepsis as both human neonatal and adult monocytes displayed similar phagocytic and intracellular killing capacity (Strunk et al., 2007). Taken together, this indicates that neutrophils play a particularly important role in the immune response during *S. epidermidis* sepsis.

While there are numerous studies dedicated to understanding the role of the innate immune response during *S. epidermidis* sepsis, research on the role of the adaptive immune response in *S. epidermidis* septic infections remains limited. As these infections are acute in nature, it is unlikely that the adaptive immune response plays a significant role. Accordingly, intravenous transfer of immunoglobulin from donors with high titers of antibodies to *S. epidermidis* or *S. aureus* failed to protect from sepsis in preterm newborns (Bloom et al., 2005; DeJonge et al., 2007).

## Conclusions

CoNS infections and in particular *S. epidermidis* pose a significant medical and economic burden to public healthcare. Furthermore, with the emergence of antibiotic resistance, treatment options are becoming limited. However, the elimination of *S. epidermidis* bacteria appears inappropriate, as they are an integral part of the beneficial microbiota on the skin and mucous membranes.

The beneficial role of *S. epidermidis* colonization in the prevention of pathogen overgrowth on the skin, based on results in mice, has been attributed to stimulation of the immune response. However, with the mouse skin model barely reflecting the situation on human skin, it remains poorly understood whether the findings are transferable to humans. Furthermore, whether there is a role of potential direct bacterial interaction between *S. epidermidis* and pathogens on the skin remains to be investigated.

The immune response to *S. epidermidis* during infection possibly is even less well understood. In chronic, biofilm-associated infection it is difficult to distinguish between effects of shear agglomeration and those mediated by specific cell surface components. During sepsis, the nature and role of pro-inflammatory cytokines and inflammation pathways in response to *S. epidermidis* remains equally poorly defined. Elucidation of these mechanisms will also provide more information on the question why neonates are particularly susceptible to *S. epidermidis* infection. Progress that has been made regarding molecular biology tools for *S. epidermidis* and CoNS in general will be of great help in these endeavors and especially assist in differentiating between direct and secondary effects. Clearly, a deeper understanding of the host immune response to these infections will be critical to the development of *S. epidermidis* vaccines and novel therapies.

## Author contributions

TN and MO wrote the manuscript. MP and TN prepared figures.

### Conflict of interest statement

The authors declare that the research was conducted in the absence of any commercial or financial relationships that could be construed as a potential conflict of interest.
